# Eco-friendly production of silver-copper nanoparticles using coconut husk and evaluation of its anti-cancer properties on human breast cancer (MDA-MB-231) cell line

**DOI:** 10.3389/fmolb.2025.1653312

**Published:** 2025-09-11

**Authors:** Atheer M. Asiri, Daoud Ali, Nawal M. Al-Malahi, Mohammed H. A. Almarzoug, Bader O. Almutairi, Saad Alkahtani, Badr A. Aldahmash, Saud Alarifi

**Affiliations:** Department of Zoology, College of Science, King Saud University, Riyadh, Saudi Arabia

**Keywords:** gAg@CuNPs, ROS, MDA-MB-231 cells, MTT assay, apoptosis

## Abstract

The biological processes using green synthesis tool is safe, environmentally friendly, non-toxic, and economical, they are more suited to manufacturing nanoparticles with sizes between 1 and 100 nm than other related techniques. Here, I have used chemical methods to create bimetallic silver copper nanoparticles (Ag@CuNPs) utilizing coconut husk. Before exposure to target cells, the characterization of green silver copper nanoparticles (gAg@CuNPs) was done by UV vis spectrophotometer, scanning electron microscope (SEM), transmission electron microscopy (TEM), DLS. The shape of gAg@CuNPs are spherical and its size is measured 43.50 ± 1.5 nm. The cytotoxic effect of g Ag-Cu NPs on human breast cancer (MDA-MB-231) cells was determined by MTT and NRU tests. The cytotoxicity of gAg@CuNPs increased in a concentration-dependent manner and it showed high cytotoxic effect on MDA-MB-231 cells at the highest concentration of NPs exposure. From the MTT result I determined the median inhibitory concentration (IC_50_) for MDA-MB-231 cells at 24 h, which came out to be 66 μg/mL of NPs. Intracellular ROS levels was elevated at higher concentration of gAg@CuNPs. Superoxide dismutase (SOD) levels was increased at 33 μg/mL and it was reduced at 45 μg/mL. Glutathione (GSH) was reduced in MDA-MB-231 cells at high concentration of gAg@CuNPs. Using JC-1 staining, the loss of mitochondrial membrane potential in control, and gAg@CuNPs exposed cell were evaluated. In MDA-MB-231 cells, maximum apoptotic cells were observed at high concentration of NPs. Caspase-3/7 activity was increased in MDA-MB-231 cells at higher concentration of NPs. The above findings highlight the significance of gAg@CuNPs as cytotoxic agent brought on by oxidative stress, which frequently useful in a number of cancer treatments.

## 1 Introduction

Biosensing platforms, immunotherapy, wound healing, food technology, waste degradation and electrochemical sensing, tissue regeneration, regenerative medicine, drug delivery and dentistry are some of the biomedical fields that are seeing new applications for nanomaterials and green-produced metal oxide nanoparticles (Saadat et al., 2020; Das et al., 2021; Dey et al., 2022; Pandey et al., 2023; Pandey et al., 2023 a; Masoud et al., 2025). Green synthesis techniques are more stable, non-toxic, cost-effective, and ecologically friendly than traditional biological, physical, and chemical processes (Mustapha et al., 2022). Nanoparticles (NPs) are defined as particles with diameters ranging from 1 to 100 nm. Considering their unique size range of 1 nm–100 nm, form, and structure, they exhibit complete uniqueness and improved properties (Jahn, 1999). Due to their application in medical science, these substances are becoming more and more common. Colloidal silver is used to make ointments, bandages, and wound dressings as well as to treat bacterial infections in exposed wounds (Abbasi et al., 2016). Antimicrobial, antifungal, antioxidant, and anti-inflammatory properties have all been applied to silver nanoparticles (AgNPs) (Sampath et al., 2022). Investigations into nanoparticle safety and exposure routes are ongoing. Despite their widespread use in nanotechnological applications, the most recent investigation revealed that there was a lack of systematic evaluation of the DNA-damaging and carcinogenic potential of bimetallic NPs (Arora et al., 2020).

In this work, I describe a synthetic approach that uses the chemical precipitation process to create bimetallic silver (Ag) and copper (Cu) nanoparticles. Compared to their constituent metals, Ag-Cu bimetallic nanoparticles have been shown to exhibit superior oxidation resistance, electrical conductivity, and catalytic performance (Hsieh and Hung, 2016). Because of their synergistic qualities, bimetallic silver copper nanoparticles (Ag@CuNPs) are used in a variety of sectors, most notably biomedicine as broad-spectrum antibiotics and possible cancer treatments (Li et al., 2018). Ag@CuNPs also have potential in electronics because of their high conductivity and are employed as catalysts for environmental applications, such as breaking down nitrophenols and other contaminants. When compared to their monometallic equivalents, their applications benefit from increased stability, decreased toxicity, and higher antibacterial activity (Li et al., 2018).

In the research of the mechanism and function of cancer cells against xenobiotic materials, human breast cancer (MDA-MB-231) cells have become a crucial model system. Because cancer therapy marks a new era marked by improvements in the specificity, efficacy, and tolerance of cancer treatments, cancer cells are of special interest in nanotoxicological and/or nanomedicine. Using inexpensive adsorbents like coconut husk and wasted impra ginseng-flavored green tea to remove harmful metals like cadmium, chomium, and lead from greywater will benefit both the environment and public health (Duwiejuah et al., 2024). Biochar made from coconut husks and wasted impra ginseng-flavored green tea are straightforward, affordable, accessible, and environmentally friendly technologies that can be readily included and upgraded for a real-world use in an aquatic environment to remove contaminants.

The harmful effects on cancer cells are influenced by particle size, composition, and related reactivity (Mirowsky et al., 2013). Increased ROS production has been linked to this observation. The overproduction of ROS in live tissue under stress is one among the several mechanisms underlying NPs-mediated toxicity. The electron transport chain is where the majority of ROS are generated, and mitochondria are the primary source of ROS in cells. The main toxicity mechanisms of Ag-Cu NPs are oxidative stress, apoptotic responses, and genotoxicity reactions. Therefore, I investigated the cytotoxicity and apoptotic properties of gAg@CuNPs on MDA cells over 24 h. Additionally, our findings will help evaluate the safety and environmental friendliness of using Ag-Cu NP in industry.

## 2 Materials and methods

### 2.1 Chemicals and reagents

The chemicals 4,5-dimethyl-2-thiazolyl)-2,5 diphenyl-2H-tetrazolium bromide (MTT), dimethyl sulfoxide (DMSO), Hoechst 33,258 fluorescent dye, 2′,7′-dichlorodihydrofluorescein diacetate (H2DCFDA), Dulbecco’s Modified Eagle Medium (DMEM), Phosphate buffered saline (PBS), fetal bovine serum (FBS), trypsin- EDTA solution 1x, etc., were purchased from Sigma-Aldrich. Other chemicals related to the current experiment were bought at local markets.

### 2.2 Synthesis of silver copper nanoparticle (gAg@CuNPs) from coconut husk

I purchased whole coconut from local market of Riyadh Saudi Arabia and separated husk from coconut and grinded into a powder by using a grinder (Ikon, China). 50 gm of coconut husk powder was mixed with 100 mL of Milli-Q deionized water in a 250 mL glass conical flask, the mixture will be put at 4 °C for 48 h before being warmed at 100 °C for 60 min. To separate the aqueous extract and remove the heavy biomaterials, it was centrifuged at 13,000 rpm for 15 min and filtered using Whatman filter paper. To prepare Ag-Cu NPs using a precipitation reaction technique, 30 mL of extract of coconut husk was mixed with 10 mL of silver chloride and coper sulfate in a 100 mL conical flask. After, it was constant mixed, the color of the extract will be changed to blackish brown as a result of the reduction of Ag and Cu ions. After the reaction was finished, the suspension was mixed for 24 h at 30 °C at 500 rpm. The precipitate material was cleaned thee times with distilled water and was dried at room temperature for 24 h and on a hot plate at 100 °C for 4 h. The black-grey powder was a green Ag-Cu NPs.

### 2.3 Physiochemical characterization of gAg@CuNPs

The gAg@CuNPs' spectrum was determined using a UV-Vis spectrophotometer (Shimadzu, Japan), and their size and shape have been evaluated using TEM (JEOL Inc., Tokyo, Japan) and scanning electron microscopy (SEM) (JEOL Inc., Tokyo, Japan), respectively. Dynamic light scattering (DLS) and a zeta sizer (Malvern, UK) were used to determine the hydrodynamic size and zeta potential, respectively.

### 2.4 Cell culture and exposure to nanoparticles

Human breast cancer (MDA-MB-231) cell lines were brought from American Type Culture Collection, Manassas, VA (ATCC). MDA-MB-231 cell lines are used in research due to their represent an easily accessible, affordable, and renewable *in vitro* model for examining the genetic and molecular aspects of the illness, simulating its course, and testing novel treatments. Cell lines were cultured in DMEM complete media that were prepared previously by adding 50 mL of preheated 10% fetal bovine serum (FBS) to 450 mL DMEM media and 5 mL of antibiotics, namely, penicillin and streptomycin (10,000 U/mL). The cells were incubated in a 5% CO2 incubator at 37 °C. MDA-MB-231 cell lines were sub-cultured 24 h before exposure to the nanoparticles. Control cells were not exposed to nanoparticles in each experiment. Stock suspension of gAg@CuNPs was prepared by suspending 10 mg NPs in 10 mL distilled water and sonicated for 10 min and then diluted it in complete DMEM media to eight experimental doses: 0, 1, 5, 10, 20, 50, 100, 150 μL/mL for further procedures.

### 2.5 MTT assay and IC_50_ determination

MTT assay is based on the ability of viable cells to convert the yellow, water-soluble MTT dye (3-(4,5-dimethylthiazol-2-yl)-2,5-diphenyltetrazolium bromide) into a purple-colored formazan crystal (Ali and Hans, 2010). This conversion occurs though the action of mitochondrial enzymes in metabolically active cells. MDA-MB-231cells (2 × 10^4^) were seeded in 96-well plate and incubated in CO_2_ incubator at 37 C for 24 h. Further on, the cells exposed to varying concentrations of gAg@CuNPs (0, 1, 5, 10, 20, 50, 100, 150 μL/mL) for a duration of 24 h (each sample was run in quintuplicate). Later, the plate was centrifuged and washed with PBS and then MTT dye solution (3 mg/mL) was added to each well, then the plate was incubated for 4 h to allow for the formation of formazan crystals by metabolically active cells. Then, formazan crystal was solubilized in dimethyl sulfoxide (DMSO) after 15-min incubation at room temperature. The optical density of the culture plate was measured at 570 nm using a microplate reader (BioTek Instruments, Winooski, VT) with the Gen5 software (version 1.09). This absorbance correlates with the amount of formazan produced, which reflects cell viability or proliferation.

The 24-h half-maximal inhibitory concentration (IC_50_) of the NPs was determined based on the MTT test result (Table 1).

**TABLE 1:** Half-maximal inhibitory concentration (IC50) and the determined concentrations of the NPs applied on the MDA-MB-231 cells.

IC50 − 24 h = 66 μg/mL of NPs	
Percentage %	Concentration
25% of IC_50_	16.5 μg/mL
50% of IC_50_	33 μg/mL
75% of IC_50_	49.5 μg/mL

### 2.6 Neutral red uptake test

The neutral red uptake (NRU) assay is a cytotoxicity assay used to assess cell viability and cell membrane integrity 9Ali et al., 2011). Upon seeding (2 × 10^4^) MDA-MB-231 cells in 96-well plate, the cells were exposed to the eight doses of gAg@CuNPs (0, 1, 5, 10, 20, 50, 100, 150 μL/mL) for 24 h (each sample was run in quintuplicate). Then, the plate was centrifuged and washed with the PBS and 100 µL of NR dye solution (2.5 mg/mL) was added to each well. The plate was Incubated for 3 h to allow the cells to uptake the NR dye. Upon completion of the 3-h incubation with NR, the media containing NR discarded from the wells and the fixative solution (0.5 mL of 37%–40% formaldehyde (0.5%) with 1 g of CaCl_2_ (1%) in 98.5 mL of d. H_2_O) was added to each well and immediately discarded to stops cellular processes and preserves the staining. Then, the destaining solution (50 mL of absolute ethanol (50%) with 49 mL of d. H_2_O and 1 mL of glacial acetic acid (1%)) was added to each well and the plate was incubated for 15 min under constant shaking. After destaining, the absorbance of the plate was read at 540 nm using a microplate reader (BioTek Instruments, Winooski, VT) with the Gen5 software (version 1.09). This absorbance correlates with the amount of NR taken up by the cells, which reflects cell viability.

### 2.7 Intracellular reactive oxygen species (ROS) generation

Upon exposure of MDA-MB-231 cells to gAg@CuNPs, ROS generation was measured to assess oxidative stress levels, which can indicate cellular damage and apoptosis. MDA-MB-231 cells (2 × 10^4^) were seeded in a 96-well black culture plate and maintained in a CO_2_ incubator at 37 C for 24 h. Subsequently, the cells were treated with gAg@CuNPs at concentrations of 16.5, 33, and 49.5 μL/mL and incubated for another 24 h, with each sample tested in quintuplicate. Afterward, the culture plate treated with the nanoparticles was incubated for 24 h. After this incubation period, DCFH-DA (2′,7′-dichlorofluorescein diacetate, 10 μM) prepared in complete culture medium was added to each well and incubated for 30 min at 37 °C. The plates were then rinsed with chilled PBS, and fluorescence intensity was measured at 485 nm excitation and 535 nm emission using a microplate reader with Gen5 software (version 1.09; Bio-Tek Instruments, Winooski, VT). The results were expressed as percentages of fluorescence intensity relative to the controls. Additionally, ROS generation was assessed though a qualitative analysis method. Briefly, MDA-MB-231 cells (5 × 10^4^) were seeded in a 6-well plate and incubated in a CO_2_ incubator at 37 °C for 24 h. After a 24-h exposure to gAg-Cu NPs, the cells were treated with DCFH-DA, which was prepared by adding 50 μL to 8 mL of complete culture medium. The plate was then incubated for 30 min, followed by a PBS wash. Subsequently, 100 μL of PBS was added to each well. Fluorescence images were captured using a fluorescence microscope fitted with a CCD cool camera (Nikon Eclipse 80i equipped with a Nikon DS-Ri1 12.7-megapixel camera).

### 2.8 Mitochondrial membrane potential (MMP)

Mitochondrial membrane potential (ΔΨ_m_) is a crucial indicator of cell health. This assay was performed though qualitative and quantitative analysis methods using JC-1 Mitochondrial Membrane Potential Assay Kit (Cayman Chemical, Item No. 10009172).

The JC-1 dye (5,5′,6,6′-Tetrachloro-1,1′,3,3′-tetraethylbenzimidazolylcarbocyanine iodide) is used to assess mitochondrial membrane potential. The principle behind using JC-1 dye is based on its ability to change colour depending on the membrane potential. In healthy cells with high mitochondrial membrane potential, JC-1 aggregates in the mitochondria and emits red fluorescence. However, in cells with low membrane potential, JC-1 remains in its monomeric form, which emits green fluorescence.

To measure the ΔΨ_m_ though a qualitative analysis method, (2 × 10^4^) MDA-MB-231 cells were seeded in 96- well black culture plate and kept in a CO_2_ incubator at 37 C for 24 h. Later, the cells treated with gAg@CuNPs (16.5, 33, and 49.5 μL/mL) were incubated for 24 h (each sample was run in quintuplicate). After 24 h, 50 µL of JC-1 dye was mixed with 10 mL complete media, and 100 µL of this mixture was added to each well, and then, the plate incubated for 30 min. The following steps were done according to the kit’s instructions. A microplate reader with the Gen5 software (version 1.09; Bio-Tek Instruments, Winooski, VT) was used to evaluate the intensity of red and green fluorescence which reflects the relative health and functionality of the mitochondria within the cells. Green fluorescence intensity (JC-1 monomers) was measured at 485 nm excitation and 535 nm emission. The red fluorescence intensity (JC-1 aggregates) was measured at 535 nm excitation and 595 nm emission. The results were expressed as the ratio of red to green fluorescence in comparison with those of the control.

A separate set of experiments was conducted to assess mitochondrial membrane potential using a qualitative analysis approach. Briefly, MDA-MB-231 cells (5 × 10^4^) were seeded on coverslips in a 6-well plate and incubated in a CO2 incubator at 37°C for 24 h. The cells were then exposed to gAg@CuNPs at concentrations of 16.5, 33, and 49.5 μL/mL for 24 h. Following this exposure, JC-1 dye solution was carefully added to each well containing the coverslips, and the plate was incubated for 30 min. After incubation, the coverslips were washed with PBS before being transferred to slides. Fluorescence images were then captured using confocal microscopy (Zeiss LSM 780 confocal microscope).

### 2.9 Glutathione (GSH) and superoxide dismutase (SOD) levels

The glutathione (GSH) and superoxide dismutase (SOD) levels were quantified following the protocol provided in the Glutathione Assay Kit (Cayman Chemical, Item No. 703002) and Superoxide Dismutase Assay Kit (Cayman Chemical, Item No. 706002), respectively. MDA-MB-231 cells (2 × 10^6^) were seeded into four T25 cell culture flasks for each assay and incubated at 37 C in a CO_2_ incubator for 24 h. Following this initial incubation, the cells were exposed to gAg@CuNPs at concentrations of 16.5, 33, and 49.5 μL/mL, with one flask serving as the untreated control. All flasks were then incubated for an additional 24 h. After the incubation period, the media was carefully aspirated, and the cells were washed with chilled PBS. For the GSH assay, GSH MES cell lysis buffer was added to each flask, while for the SOD assay, cells were harvested in 20 mM HEPES buffer containing 1 mM EGTA, 210 mM mannitol, and 70 mM sucrose (pH 7.2). The cells were then harvested using a rubber policeman cell scraper, and the suspensions were collected into small glass tubes on ice. The cell suspensions were sonicated on ice for 5 minutes, transferred to 15 mL conical tubes, and centrifuged at 4 C for 15 min at 10,000 × g. A 500 µL aliquot of the supernatant was then transferred into small Eppendorf tubes and stored at −80 °C for subsequent analysis.

Total GSH and SOD concentrations were determined according to the respective manufacturer’s instructions. Absorbance for GSH was measured at 410 nm, while absorbance for SOD was measured at 450 nm, both using a microplate reader equipped with Gen5 software (version 1.09; Bio-Tek Instruments, Winooski, VT).

### 2.10 Measurement of caspase 3/7 activity

Caspase-3 and caspase-7 are critical executioner proteases in the apoptotic pathway, playing a pivotal role in the final stages of programmed cell death. Upon activation by initiator caspases, these enzymes cleave a broad range of substrates, leading to the hallmark features of apoptosis. To assess the execution of apoptosis following the exposure of MDA-MB-231 cells to gAg@CuNPs, caspase-3/7 activity was evaluated using Caspase-3/7 Fluorescence Assay Kit (Cayman Chemical, Item No. 10009135).

In summary, MDA-MB-231 cells were seeded at a density of 8 × 10^4^ cells per 100 μL of culture medium in 96-well plates. The cells were then exposed to varying concentrations of gAg@CuNPs (16.5, 33, and 49.5 μL/mL) for 24 h. Following the exposure, the assay procedure began with centrifugation of the plate at 3,000 rpm for 5 min, after which the supernatant was carefully removed. Subsequently, 200 μL of assay buffer was added to each well, and the plate was centrifuged again at 3,000 rpm for 5 min before the supernatant was discarded. Each well then received 100 μL of cell-based assay lysis buffer, and the plate was incubated on a shaker for 30 min at room temperature. After incubation, the plate was centrifuged at 3,000 rpm for 10 min, and 90 μL of the supernatant was transferred to a black 96-well plate. To the appropriate wells, 10 μL of assay buffer and 10 μL of caspase-3/7 inhibitor solutions were added. Finally, 100 μL of the caspase-3/7 substrate solution was introduced to each well, and the plate was incubated for 90 min at 37 °C. Fluorescence intensity was then measured using a microplate reader with Gen5 software (version 1.09; Bio-Tek Instruments, Winooski, VT), with excitation at 485 nm and emission at 535 nm.

### 2.11 Immunofluorescence assay (bax, p53, and caspase 3 expression)

Immunofluorescence was employed to investigate the potential cell death pathways induced by gAg@CuNPs, specifically though the expression of bax, p53, and caspase 3. MDA-MB-231 cells were seeded onto coverslips placed within each well of a 6-well plate at a density of 8 × 10^4^ cells per well and incubated under optimal conditions for 24 h. Subsequently, the cells were exposed to varying concentrations of gAg@CuNPs (16.5, 33, and 49.5 μL/mL) for 24 h. Following exposure, the cells were washed twice with chilled PBS and then fixed with 1 mL of cold paraformaldehyde. After fixation, the cells were washed with cold Tris Buffered Saline with Tween 20 (TBST) to minimize non-specific protein-protein interactions. To prevent non-specific antibody binding, 1 mL of cold blocking buffer was added, and the plates were incubated in a hood for 30 min. The cells were then washed thee times with TBST. Primary antibodies (anti-bax, anti-p53, and anti-caspase 3) from Cayman Chemical were prepared at a dilution of 3 µL antibody to 8 mL TBST and added to the culture plates, which were then covered with aluminum foil and incubated at 4 °C in the dark for 24 h. After incubation, the cells were washed thee times with cold TBST. In the dark, 200 µL of secondary antibody (Goat Anti-Mouse IgG H&L, Alexa Fluor® 488, Abcam, Cat# ab150113) was added, followed by the addition of 200 µL of DAPI for nuclear staining. The plates were incubated in the dark for 1 hour. Finally, the coverslips were gently washed with TBST, and fluorescence images were captured using confocal microscopy (Zeiss LSM 780 confocal microscope).

### 2.12 Statistical analysis

Results are expressed as mean ± standard error (SE). Statistical analyses were performed using the SPSS 26.0 (IBM) software package. Group comparisons were performed using one-way analysis of variance (ANOVA). Differences at *p* ≤ 0.05 considered significant. The Tukey post-test was carried out for intergroup comparisons. SPSS 26.0 (IBM) software package used to create graphical data representation.

## 3 Results

### 3.1 Characterization of gAg@CuNPs

**FIGURE 1:**
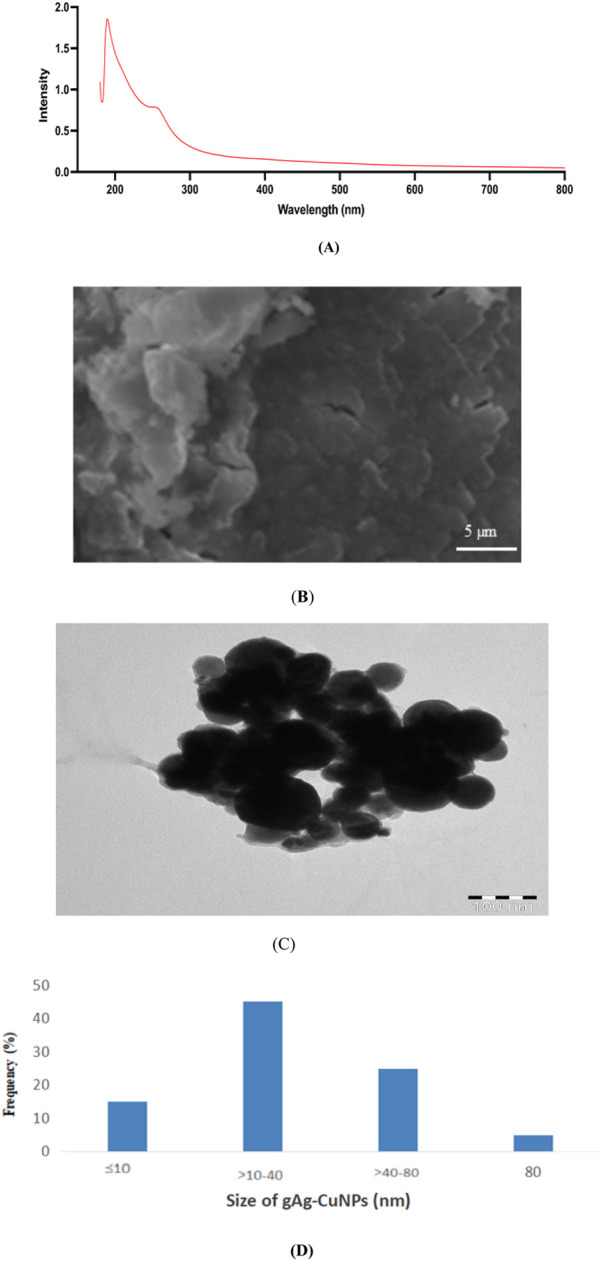
Characterization of gAg@CuNPs. **(A)** UV spectrum and **(B)** image of gAg@CuNPs by scanning electron microscope. **(C)** Image of gAg@CuNPs by transmission electron microscope (JEM 1011). **(D)** Distribution of gAg@CuNPs in water suspension.

Figure 1A represents the UV vis spectrum of gAg@CuNPs and the SEM result confirmed the presence of Ag, Cu in gAg@CuNPs and of other elements (Figure 1B). Figure 1C showed the TEM image of gAg@CuNPs. The normal size of gAg@CuNPs is 42.5 ± 1.5 nm (Figure 1D). DLS and zeta potentials were used to confirm the particle size and stability of gAg@CuNPs in water suspension. The size of gAg@CuNPs was determined at 189 ± 2.5 nm zeta potential of the NPs in aqueous solution was −0.86 mV.

### 3.2 Cytotoxicity

**FIGURE 2:**
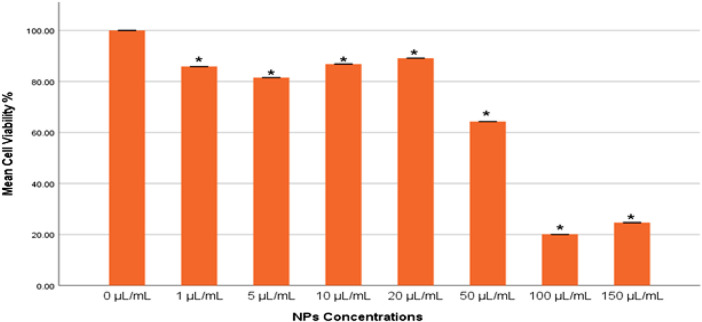
Cytotoxicity of gAg@CuNPs in MDA-MB-231 cells over 24 h, as evaluated by MTT assays. Values are means ± SE of six experiments. The asterisk (*) indicates a statistically significant p < 0.05 compared with the control group.

The cytotoxic effects of the gAg@CuNPs on MDA-MB-231 cells were assessed using the MTT assay. The results demonstrated that gAg@CuNPs induced cytotoxicity in a dose-dependent manner (Figure 2). Cells treated with gAg@CuNPs exhibited significantly (*p* < 0.05) higher rates of cell death compared to controls. Notably, a pronounced toxic effect was observed in MDA-MB-231 cells at a concentration of 100 μg/mL.

**FIGURE 3:**
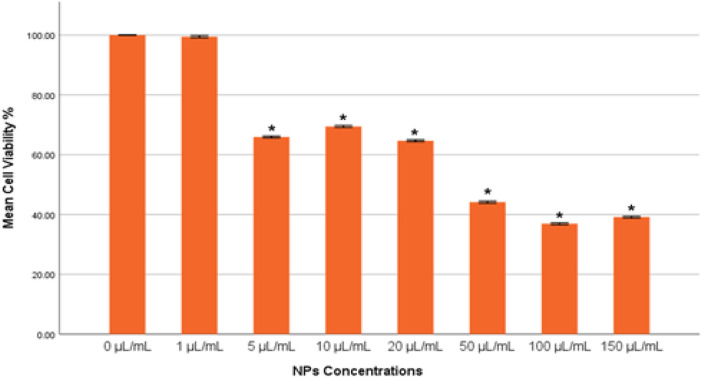
Cytotoxicity of gAg@CuNPs in MDA-MB-231 Cells over 24 h, as evaluated by NRU assays. Values are means ± SE of six experiments. The asterisk (*) indicates a statistically significant p < 0.05 compared with the control group.

The integrity of lysosomes in MDA-MB-231 cells exposed to gAg@CuNPs was evaluated using the neutral red uptake (NRU) assay. The results from the NRU assay closely aligned with those obtained from the MTT assay, displaying a consistent trend (Figure 3). The NRU assay indicated no statistically significant differences (*p* > 0.05) in cell toxicity between the control group and the treated with 1 μg/mL of gAg-Cu NPs. However, a significant decrease in cell viability (*p* < 0.05) was observed as the concentration of gAg@CuNPs increased.

### 3.3 Reactive oxygen species (ROS)

**FIGURE 4:**
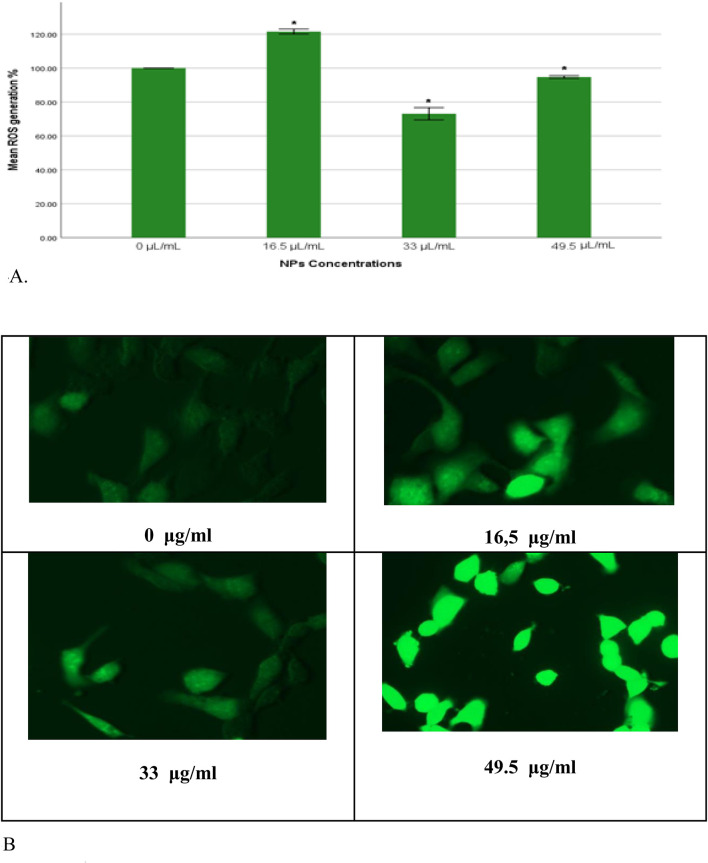
**(A)** Percentage of intracellular ROS production in MDA-MB-231 cells exposed to gAg@CuNPs over 24 h. Values are represented as means ± SE. The asterisk (*) indicates a statistically significant p < 0.05 compared with the control group. **(B)** Production of ROS in MDA-MB-231 cell line after exposure to various concentrations of gAg-Cu NPs. Fluorescence images were captured using a fluorescence microscope fitted with a CCD cool camera (Nikon Eclipse 80i equipped with a Nikon DS-Ri1 12.7-megapixel camera).

The results from both quantitative and qualitative analyses demonstrated consistent trends in reactive oxygen species (ROS) production upon exposure to gAg-Cu NPs. Quantitatively, a significant increase in ROS production (*p* < 0.05) was observed in the group treated with 16.5 μL/mL of NPs compared to the control untreated cells (Figure 4A). Conversely, a significant decrease (*p* < 0.05) in ROS levels was detected in the groups exposed to 33 and 49.5 μL/mL of NPs when compared to the control group.

In parallel, qualitative analysis using fluorescence microscopy provided visual confirmation of these findings. MDA-MB-231 cells were imaged following nanoparticle exposure using a fluorescence microscope. The fluorescence intensity of DCFH-DA, which indicates ROS levels, mirrored the quantitative results, with an evident increase in fluorescence intensity in the 16.5 μL/mL group and a corresponding decrease in the 33 and 49.5 μL/mL groups (Figure 4B).

### 3.4 Mitochondrial membrane potential (MMP)

**FIGURE 5:**
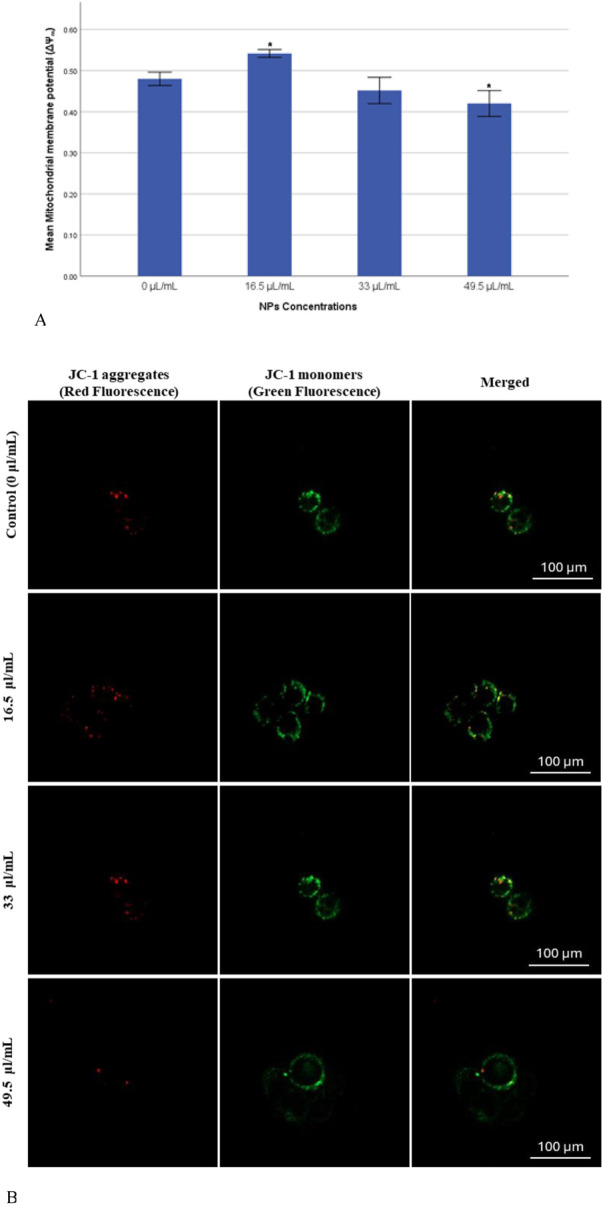
**(A)** Mitochondrial membrane potential (ΔΨ_m_) of MDA-MB-231 Cells exposed to gAg@CuNPs over 24 h. Values are means ± SE of six experiments. The asterisk (*) indicates a statistically significant p < 0.05 compared with the control group. **(B)** JC-1 staining pattern indicates the mitochondrial membrane potential in MDA-MB-231 cells exposed to gAg@CuNPs over 24 h. The cells imaged by fluorescence confocal microscopy (Zeiss LSM 780 confocal microscope). Cells were labeled by JC-1 to visualize mitochondria. Fluorescence was registered using excitation at 488 nm and adjusting the emission of confocal microscopy for J-monomers (visible as green) and J-aggregates (visible as red).

The mitochondrial membrane potential (ΔΨ_m_) was measured to assess the progression of apoptosis, as its decline is commonly recognized as an early indicator of apoptotic activity though the intrinsic pathway, which is a critical mechanism of programmed cell death. In this study, a significant reduction (*p* < 0.05) in JC-1 fluorescence ratio (J-aggregates: J-monomers) was observed in the group exposed to a dose of 49.5 μL/mL, indicating a marked induction of apoptosis (Figure 5A). In contrast, the group exposed to a dose of 33 μL/mL exhibited a reduction in JC-1 fluorescence ratio that was not statistically significant (*p* > 0.05). Notably, the group treated with 16.5 μL/mL demonstrated a significant surge (*p* < 0.05) in ΔΨ_m_, suggesting a potential resistance or alternative cellular response to this dose.

In parallel with the previous findings, the current study also assessed mitochondrial membrane potential (MMP) to detect early apoptotic changes in MDA-MB-231 cells using JC-1 as a fluorescent probe. The formation of fluorescent dye within the mitochondria was visualized though confocal microscopy. The confocal microscopy images revealed results that closely align with those obtained from the quantitative assay (Figure 5B), further validating the observed trends in ΔΨ_m_ across different treatment groups.

### 3.5 SOD and GSH enzymes in MDA-MB-231 cells

**FIGURE 6:**
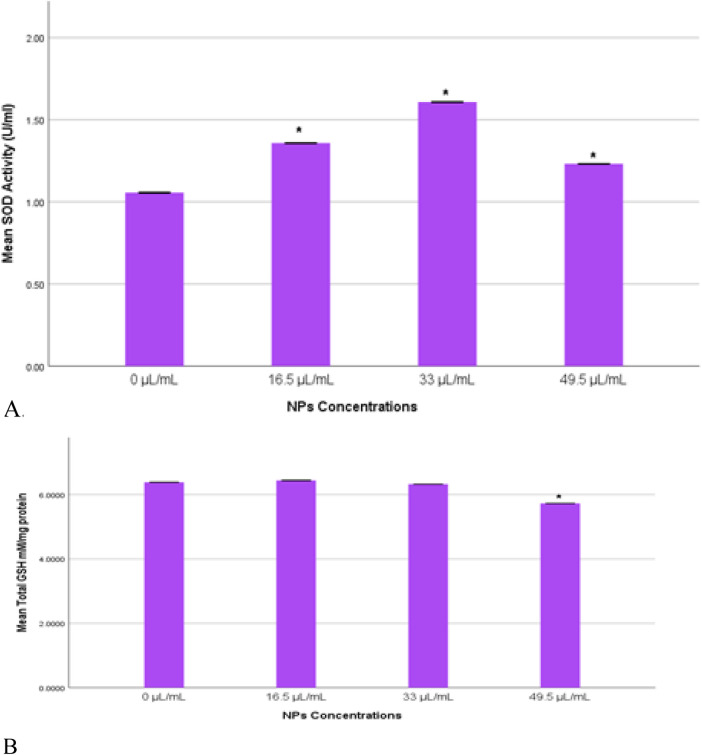
**(A)** SOD activity (U/mL) in MDA-MB-231 cells exposed to gAg@CuNPs over 24 h. Values are represented as means ± SE. The asterisk (*) indicates a statistically significant p < 0.05 compared with the control group. **(B)** Total GSH (mM/mg) in MDA-MB-231 cells exposed to gAg@CuNPs over 24 h. Values are represented as means ± SE. The asterisk (*) indicates a statistically significant p < 0.05 compared with the control group.

Oxidative stress was assessed by measuring the levels of SOD and GSH enzymes in MDA-MB-231 cells exposed to Ag-Cu coconut nanoparticles. Both GSH and SOD levels were quantified and statistically compared to those in control cells. A significant increase (*p* < 0.05) in SOD levels was observed across all experimental groups compared to the control (Figure 6A). Notably, the group exposed to 33 μL/mL exhibited the highest SOD enzyme activity. The total GSH levels (mM/mg) showed no significant changes (*p* > 0.05) across most groups. However, a significant reduction (*p* < 0.05) in total GSH levels was observed in cells exposed to 49.5 μL/mL of Ag-Cu coconut nanoparticles (Figure 6B).

### 3.6 Caspase 3/7 activity

**FIGURE 7:**
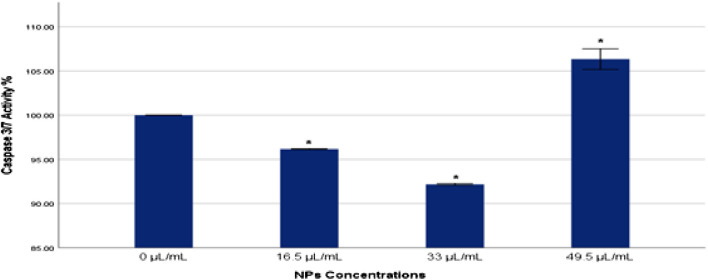
Caspase 3/7 activity % in MDA-MB-231 cells exposed to gAg@CuNPs over 24 h. Values are represented as means ± SE. The asterisk (*) indicates a statistically significant p < 0.05 compared with the control group.

Caspase 3/7 activity plays a critical role in the execution phase of apoptosis, serving as key effectors in the apoptotic signaling pathway. In the current study, caspase 3/7 activity was evaluated to gauge the extent of apoptosis in MDA-MB-231 cells exposed to Ag-Cu coconut nanoparticles (Figure 7). A significant increase (*p* < 0.05) in caspase 3/7 activity was detected in cells exposed to a higher concentration (49.5 μL/mL) of these nanoparticles compared to the control group. Conversely, cells exposed to lower concentrations of Ag-Cu coconut nanoparticles (16.5 and 33 μL/mL) showed a significant decrease (*p* < 0.05) in caspase 3/7 activity.

### 3.7 Immunofluorescence assay (bax, p53, and caspase three expression)

**FIGURE 8:**
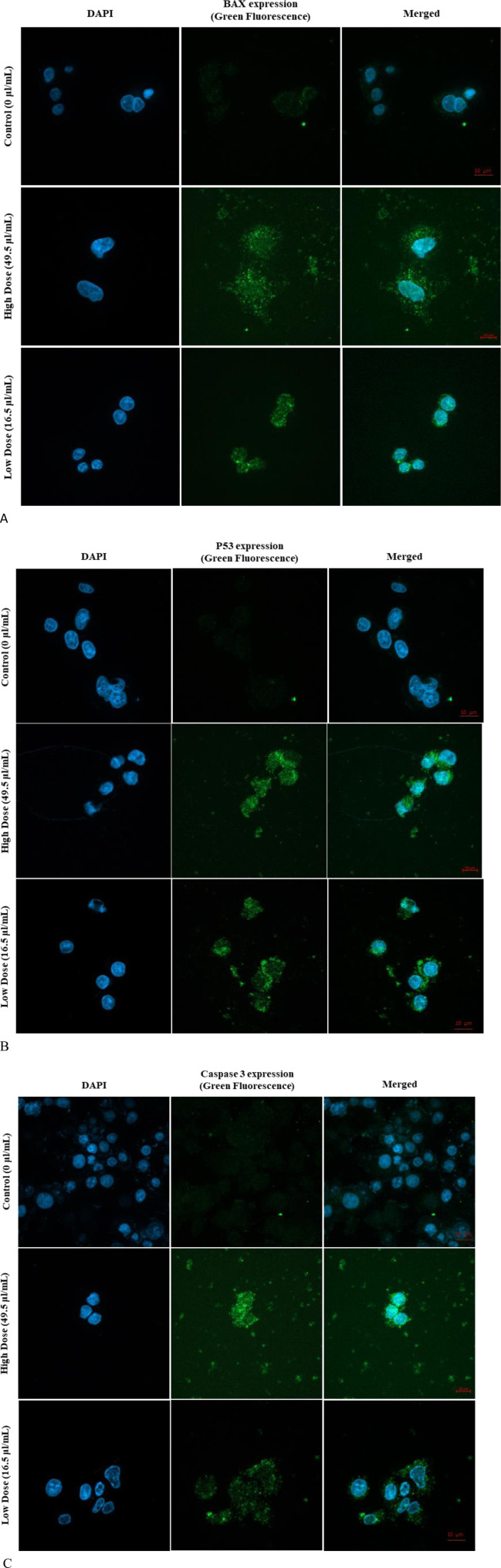
**(A)** Expression of Bax in MDA-MB-231 cells following 24-h exposure to Ag-Cu coconut nanoparticles. The image shows DAPI-stained nuclei (blue fluorescence) and Bax apoptotic protein expression (green fluorescence). **(B)** Expression of p53 in MDA-MB-231 cells following 24-h exposure to Ag-Cu coconut nanoparticles. The image shows DAPI-stained nuclei (blue fluorescence) and P53 apoptotic protein expression (green fluorescence). **(C)** Expression of caspase three in MDA-MB-231 cells following 24-h exposure to Ag-Cu coconut nanoparticles. The image shows DAPI-stained nuclei (blue fluorescence) and caspase three apoptotic protein expression (green fluorescence).

The expression levels of key apoptotic proteins, including bax, p53, and caspase 3, in MDA-MB-231 cells exposed to Ag-Cu coconut nanoparticles were qualitatively assessed using immunofluorescence techniques (Figures 8A–C). This analysis employed two distinct filter channels: DAPI, which emits blue fluorescence to visualize the cell nuclei, and FITC, which emits green fluorescence to specifically highlight the presence and localization of apoptotic proteins within the cells. Referring to Figures 8A–C, the expression levels of bax, p53, and caspase three were noticeably higher in cells exposed to Ag-Cu coconut nanoparticles compared to unexposed cells, which showed no detectable expression of these proteins.

## 4 Discussion

One method that is strongly dominating the manufacturing of nanoparticles is the green synthesis technique. When compared to alternative techniques, the process is a cost-effective, environmentally benign, and non-toxic instrument. The amaranthaceous family includes the plant used for this study, which is widely distributed in tropical and subtropical areas worldwide. Ayurveda places great weight on it because of its rich phytochemical makeup, which includes flavonoid glycosides, terpenoids, alkaloids, sterols, and polyphenols. It is used for a number of medical purposes, including antibacterial, antiasthmatic, anti-urolithiasis, and antihyperglycemic ones. Although coconut husk is generally thought to be non-toxic, processing may cause some toxicity and increase the likelihood that it contains specific microorganisms. The husk fibers are softened by the retting process, which can contaminate water and possibly introduce dangerous bacteria like *Salmonella*. The UV-visible spectrum, SEM, TEM, and DLS techniques were used to examine the characteristic properties of the produced bimetallic gAg@CuNPs. A flexible and environmentally friendly method of treating wastewater, coconut husk-based biosystems efficiently remove pathogens, lower BOD and COD, and get rid of nitrogen, phosphorus, and other contaminants. They can be used as a bio-adsorbent, a biofilm supports medium, or anaerobic filters, among other configurations (Brown et al., 2025). Their use in cancer treatment is complicated by the fate and behavior of gAg@CuNPs in biological and environmental systems, which rely on functional characteristics like size, shape, surface charge, and dispersity. Very tiny NPs have been shown to be broken down by the liver and hepatocytes and then eliminated in urine and stool. One important effect of the widespread use of nano-based materials is that humans are unaware of the dangers of being exposed to nanoparticles, which can enter the bodies of humans and animals though a variety of pathways. Because of their size-dependent characteristics, nanoparticles have a significant surface area in relation to their mass. They are useful in a variety of applications, such as study as *in vitro* models for human breast cancer cells, due to this trait and their capacity to change size in different media (Hoshyar et al., 2016). In this work, we used various methodologies to investigate how gAg@CuNPs affected MDA-MB-231 cells. gAg@CuNPs has been selected for this inquiry in order to investigate its impact on MDA-MB-231 cells growth inhibition, genotoxic responses, oxidative stress, and apoptotic cell death. The findings of dynamic light scattering research revealed the size distribution of gAg@CuNPs. Using TEM, the size of gAg@CuNPs was found to be 42.5 ± 1.5 nm. However, MTT and NRU tests were used to assess the viability of MDA-MB-231 cells after they were exposed to gAg@CuNPs. The MTT assay is based on the reduction of water-soluble tetrazolium salt into an insoluble formazan by the mitochondrial dehydrogenase enzyme, which is only active in living cells (Alarifi et al., 2016). The MTT data indicates that growth was suppressed as a result of many disturbances in the cells' metabolic capacity. Following a 24-h exposure to gAg@CuNPs, MDA-MB-231 cells' cell viability was significantly decreased, and their 50% inhibition of cell growth (IC50 value) was 66 μg/mL. Strangely, there has not been a thorough examination of MDA-MB-231 cell growth, gAg@CuNPs cytotoxicity, ROS generation, or apoptotic protein expression studies. These results imply that the specific mechanism of gAg@CuNPs toxicity is most likely oxidative stress. According to reports, this is connected to NPs' cytotoxic effects on both cancerous and healthy cells (Paget et al., 2015). Our findings showed that the combination of Ag and Cu constitutes a specific dangerous entity that produces higher ROS, which is consistent with the findings of various earlier studies (Paget et al., 2015). Nanomaterials are frequently genotoxic because oxidative stress brought on by an excess of ROS is frequently closely associated with oxidative damage to proteins and DNA. Our results indicate that the combination of Ag and Cu NPs improves their capacity to produce ROS. The results showed a significant increase in ROS as a result of the cytotoxicity when comparing the ROS level to the gAg@CuNPs that exposed both cells. In human cell culture models, coconut husk extract is cytotoxic, affecting HeLa cells in particular by causing apoptosis (programmed cell death) (Kushmitha et al., 2017). The extract also appears to downregulate the Bcl2 gene, which is essential in inhibiting apoptosis, providing a mechanism for the observed cell death. However, other studies, using different cell lines and extracts, have found no significant cytotoxicity. Therefore, the toxicity of coconut husk extract is dependent on the specific extract, cell type, and concentration used (Lima et al., 2015).

According to recent studies, gAg@CuNPs increased ROS and deceased GSH levels, decreased the viability of MDA-MB-231 cell lines, and increased SOD levels. One source of mitochondrial injury is ROS generated when the mitochondrial membrane is physically damaged. The disturbed oxidant balance may result from either increased production of ROS or a weakened antioxidant defense as a result of continuous exposure to nanomaterials (Pathak et al., 2015). Our results are consistent with previous research showing that As_2_O_3_ dosages that cause BEAS-2B cells to undergo apoptosis lead to apoptotic cells exhibiting significantly greater levels of gene expression (Tang et al., 2021). Though the upregulation of p53, caspase-3/7, and bax expression, gAg@CuNPs evidently induced apoptosis in MDA-MB-231 cells. The negative effects of Ag-Cu NPs may be lessened by the anti-cytotoxic qualities of coconut husk. We found that cells that were simply exposed to high concentrations of gAg@CuNPs experienced a higher level of apoptosis than the control.

## 5 Conclusion

For the first time, Ag-Cu NPs have been made using the green synthesis method with an aqueous extract of coconut husk. An experiment was carried out in MDA-MB-231 using gAg-Cu NPs. The size, shape, content, and generation of Ag-Cu NPs were confirmed by SEM, TEM, D, and DLS analyses. Following a notable apoptosis, Ag-Cu NPs showed cytotoxic effect when added to MDA-MB-231 cells. The green techniques that are currently available are simple to apply, quick, non-toxic, safe for the environment, and beneficial for medical uses.

## Data Availability

The original contributions presented in the study are included in the article/supplementary material, further inquiries can be directed to the corresponding author.
